# MLKL as an emerging machinery for modulating organelle dynamics: regulatory mechanisms, pathophysiological significance, and targeted therapeutics

**DOI:** 10.3389/fphar.2025.1512968

**Published:** 2025-02-25

**Authors:** Yang Wang, Wei Wei, Yu Zhang, Jingrong Miao, Xiaofeng Bao, Chunfeng Lu

**Affiliations:** School of Pharmacy, Nantong University, Nantong, Jiangsu, China

**Keywords:** MLKL, organelle dynamics, disease target, small-molecule chemicals, natural products

## Abstract

Mixed lineage kinase domain-like protein (MLKL) is a pseudokinase featured by a protein kinase-like domain without catalytic activity. MLKL was originally discovered to be phosphorylated by receptor-interacting protein kinase 1/3, typically increase plasma membrane permeabilization, and disrupt the membrane integrity, ultimately executing necroptosis. Recent evidence uncovers the association of MLKL with diverse cellular organelles, including the mitochondrion, lysosome, endosome, endoplasmic reticulum, and nucleus. Thus, this review mainly focuses on the regulatory functions, mechanisms, and targets of MLKL in organelles rather than necroptosis and summarize the medical significance in multiple diseases. On this basis, we conclude and analyze the current progress and prospect for the development of MLKL-related drugs, from natural products, small-molecule chemical compounds, to proteolysis-targeting chimera. This review is aimed to propel the development of MLKL as a valid drug target and the discovery of novel MLKL-related drugs, and promote their further applications.

## 1 Introduction

Mix linkage like-domain protein (MLKL) is a pseudokinase featuring a kinase-like structure but lacking the catalytic activity (Kung and Jura, 2019; Boudeau et al., 2006). Its canonical function is to serve as the executor of the necroptotic cell death signaling (Zhang et al., 2016; Johnston and Wang, 2018; D'Arcy, 2019; He et al., 2024). In response to stimuli, tumor necrosis factor (TNF) for example, MLKL is recruited by receptor-interacting protein kinases (RIPK1 and RIPK3) and phosphorylated, then oligomerizes, anchors in and disrupts the plasma membrane, triggering the lytic cell death (Johnston and Wang, 2018). In addition to plasma membrane, MLKL has recently been discovered in various organelles, such as the mitochondrion, endoplasmic reticulum, and lysosome, where it may regulate various pathophysiological events (Martens et al., 2021). In this review, we summarize the molecular structure and characteristics of MLKL, and conclude its non-canonical biological functions in different organelles, emphasize its significance in disease pathogenesis, and propose multiple MLKL-related drug candidates with therapeutic potential, aiming to consolidate the medical significance of MLKL and promote the development and application of related drugs.

## 2 The molecular structure of MLKL

MLKL is constituted by a N-terminal four-helix bundle domain (4HBD), a C-terminal pseudokinases domain (PsKD), and a two-helix linker known as the “brace” helices (Murphy et al., 2013).

### 2.1 The 4HBD

The N-terminal 4HBD is as the only-known ‘killer’ domain in vertebrates with the function of membrane permeabilization, similar to N-terminal HeLo-like domain (HELL) of the fungal protein HELLP (Johnston and Wang, 2018; Murphy, 2020; Tanzer et al., 2016). Structurally, 4HBD is evolutionally conserved among species, containing four helices (α1–α4) where α2 and α4 helices interacting with the N-terminal region of the first brace helix (Petrie et al., 2017). In MLKL, the death-inducing core within 4HBD resides in two phospholipid-binding clusters of residues (R105/D106 and E109/E110 in mice, D107/E111 in human) in the α4 helix (Hildebrand et al., 2014; Petrie et al., 2018; Su et al., 2014; Quarato et al., 2016). However, there is a little difference in the pro-necroptotic machinery between mice and human MLKL, where mouse 4HBD is sufficient for necroptotic signaling transduction but human MLKL requires ectopic oligomerization prior to phospholipid binding (Tanzer et al., 2016). The other difference is the C86 connecting α3 and α4 helices responsible for necroptosis in human but not exist in mice (Murphy et al., 2013).

### 2.2 The PsKD

The C-terminal PsKD resembles a protein kinase domain-like structure, featuring a bilobal structure with a larger C-lobe of α-helices, a smaller N-lobe of five antiparallel β-strands, and one α-helix called αC-helix (Murphy et al., 2013; Murphy et al., 2014). Most conventional protein kinases share a common N-lobe feature of three conserved motifs-VAIK (Val-Ala-Ile-Lys) for ATP anchoring, HRD (His-Arg-Asp) for regulating catalytic activity, and DFG (Asp-Phe-Gly) for magnesium binding (Kung and Jura, 2019). However, MLKL orthologues only contain the lysine (K219 in mouse, K230 in human) of the VAIK motif in the N-lobe β3-strand but lack the catalytic loop HRD motif (named as HGK in human, HRN in mouse) and the DFG motif (Czabotar and Murphy, 2015), providing MLKL with the capacity of nucleotide binding but no residues for phosphoryl transfer as well as no catalytic activity (Zhan et al., 2021). The PsKD of both mice and human can bind with ATP, ADP, or AMP-PNP, but only without cations. However, differing from the significance of the lysine (K219) of the VAIK motif in mouse MLKL to nucleotide binding, the homologous lysine (K230) in human MLKL is dispensable for this binding (Murphy et al., 2014). Alternatively, human MLKL has a HGK motif in the catalytic loop, where K331 assists in ATP binding (Petrie et al., 2017). However, the role of ATP binding in the regulation of MLKL remains largely unknown, especially whether ATP binding switches on MLKL, and if so, whether the structural differences between mouse and human MLKL PsKD contribute to the different switching mechanisms.

The αC-helix in the PsKD of MLKL is structurally different from the classical one. In mouse MLKL, an unclassical αC-helix (S340–I346) occupies the traditional αC-helix position, hindering the glutamate (E239) from the catalytic lysine (K219) of the VAIK motif; instead, K219 interacts with Q343. Disruption of the K219:Q343 interaction causes ATP unbinding and necroptosis (Murphy et al., 2013), which means that these two sites are critical for the transition of MLKL status. Unlike mouse MLKL, the PsKD of human MLKL adopts a typical “closed” conformation associated with active protein kinases, with the glutamate (E250) in the αC-helix forming an ion pair with the lysine of the N-lobe β3-strand (Murphy et al., 2014).

Functionally, the PsKD mainly serves as the conformational switch of the 4HBD in MLKL (Petrie et al., 2017; Davies et al., 2018). In normal conditions, the PsKD anchors the 4HBD and keeps MLKL in dormancy. When phosphorylated by RIPK3 in specific sites (S345, S347, and T349 in mouse, T357 and S358 in human), the PsKD undergoes a conformational alternation and release 4HBD for subsequent activation (Martens et al., 2021).

### 2.3 The two-helix linker

A two-helix brace is a linker connecting the 4HBD and the PsKD (Davies et al., 2018). In addition to linking, more important is that the brace plays an active role in MLKL activation. On the one hand, the brace is essential for the oligomerization. The truncations in the second brace helix of mouse MLKL (residues159–169) impair homotrimer assembly; alanine-scanning mutagenesis identified critical driving factors as E161/I162 and T165/L166, which when mutated cause a deficiency of larger molecular weight complexes (Davies et al., 2018). This aligns with a previous study, where recombinant human MLKL with truncations (residues 2–154 or 1–140) fail to form oligomers without the brace region (Su et al., 2014; Quarato et al., 2016). This function of the brace region does not change with its sequence polymorphism across species, despite that the brace of human MLKL includes 9 amino acids, longer than that of mouse due to an insertion in the first brace helix (Petrie et al., 2017). On the other hand, the first brace helix and the adjacent interhelix loop propagate the activation signal from the PsKD to the executive 4HBD (Petrie et al., 2017).

## 3 Diversified functions and regulations of MLKL in organelles

### 3.1 MLKL in the mitochondrion

The mitochondrion is a phospholipid membrane-coated organelle responsible for the regulation of energy metabolism, redox balance, ion storage and exchange, and cell survival and death (Luo et al., 2020; Tian et al., 2022; Cheng Q. et al., 2021; Yan et al., 2022). Recent studies have found that MLKL anchors not only the plasma membrane but also the mitochondrial membrane (Wang et al., 2014), affecting mitochondrial homeostasis (Wang et al., 2012; Zhang S. L. et al., 2019; Xu J. et al., 2023; Srivastava et al., 2022). When activated by pro-necroptotic signal, MLKL assembles and forms an oligomer with positively charged patches on its surface. This characteristic makes MLKL prone to be attracted by cardiolipin, a negatively charged lipid on the mitochondrial membrane, and to approach, bind to, and directly damage the mitochondrial membrane (Wang et al., 2014). Nevertheless, the cation channels on the mitochondrial membrane formed by activated MLKL may allow endoplasmic reticulum-sourced Mg^2+^ passing through (Xia et al., 2016; Jiang et al., 2023). To balance the shuttling of Mg^2+^ between the endoplasmic reticulum and the mitochondrion and to increase Mg^2+^ entry into the mitochondrion is beneficial to the limitation of mitochondrial reactive oxygen species (ROS) production (Jiang et al., 2023). Thus, whether MLKL activation and translocation to the mitochondrial membrane is conducive in specific conditions remains to be revealed.

Several other factors are involved in indirectly intermediating the mitochondrion-regulatory signal from MLKL (Wang et al., 2012; Zhang S. L. et al., 2019; Srivastava et al., 2022). For example, MLKL phosphorylated by RIPK1/RIPK3 can bind to and activate phosphoglycerate mutase family member 5 (PGAM5), a mitochondrial two-histidine phosphatase (2H-phosphatase) located on the inner and outer mitochondrial membranes, leading to the recruitment and dephosphorylation of mitochondrial fission factor dynamin-related protein 1 (Drp1) and causing mitochondrial fragmentation at an early phase during necroptosis execution (Wang et al., 2012; Yu et al., 2020). Activated PGAM5 also can phosphorylate cyclophilin D (CypD), a mitochondrial peptidyl-prolyl cis-trans isomerase controlling the switching-on and -off of mitochondrial permeability transition pore (MPTP). Phosphorylated CypD binds to the inner mitochondrial membrane, promoting MPTP opening, mitochondrial matrix content release, and amplifying the necroptotic signal (Zhang S. L. et al., 2019; Amanakis and Murphy, 2020; Li et al., 2020). Another mechanism underlying MLKL-induced MPTP opening is that the translocation of MLKL to the mitochondrion triggers mitochondrial ROS production, which then activates calcium/calmodulin-dependent protein kinases-II (CaMKII) and thereby phosphorylates CypD (Srivastava et al., 2022; Hu C. et al., 2020; Yu et al., 2019). MLKL also participates in the regulation of mitochondrial dynamics (Xu J. et al., 2023; Jiang et al., 2023). Loss of MLKL can promote the interaction between autophagy-related proteins LC3B and p62, accelerate the clearance of damaged mitochondria, and mitigate oxidative damage (Xu J. et al., 2023), which suggests a regulatory role of MLKL in mitophagy.

**FIGURE 1:**
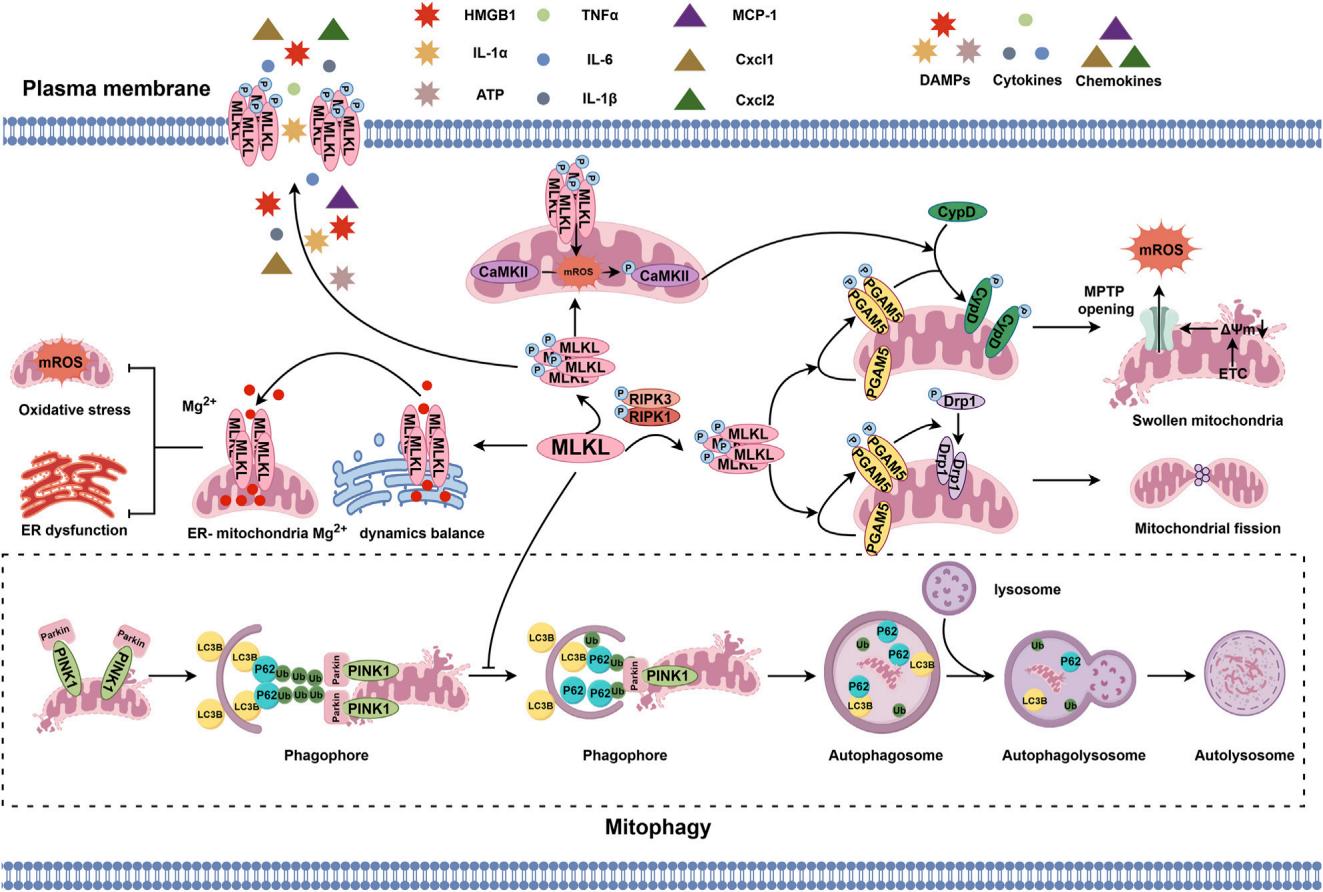
MLKL-regulated pathophysiological events in the mitochondrion. MLKL phosphorylated by the RIPK1/RIPK3 axis can bind to and phosphorylate PGAM5, which then recruits and dephosphorylates Drp1 to initiate mitochondrial fission or phosphorylates CypD to induce MPTP opening and disrupt mitochondrial homeostasis. In addition, the translocation of p-MLKL to the inner and outer mitochondrial membranes and subsequent activation of CaMKII by ROS also promote CypD phosphorylation and MPTP opening. However, the cation channels formed by MLKL on the mitochondrial and endoplasmic reticulum membrane forms are selectively permeable to Mg^2+^, which helps with stabilizing the transportation of Mg^2+^ between the mitochondrion and endoplasmic reticulum and maintaining the biological balance of both organelles. Moreover, MLKL can inhibit PINK1-mediated mitophagy by reducing LC3B and arresting p62.

Overall, MLKL functions in the mitochondrion via various mechanisms (Figure 1), thus, further explorations on the regulation of MLKL in the mitochondria and its consequence to mitochondrion dysfunction-related diseases are of great significance.

### 3.2 MLKL in the lysosome

The lysosome is a single-membrane organelle enveloping various hydrolytic enzyme, cathepsins, for example, for degrading exogenous and endogenous macromolecules (Zhang et al., 2021; Nie et al., 2024). Being activated by the upstream necroptotic signal RIPK1/RIPK3, MLKL can aggregate on the lysosomal membrane, leading to damaged lysosomal integrity and increased lysosomal membrane permeabilization (LMP); under this condition, cathepsin B (CTSB) can be leaked into the cytosol, cleave essential proteins for cell survival, and eventually trigger necroptosis (Liu S. et al., 2024). MLKL can also be recruited by activated BAX, a pro-apoptotic protein, and then translocate to the lysosome membrane (Dong et al., 2023).

**FIGURE 2:**
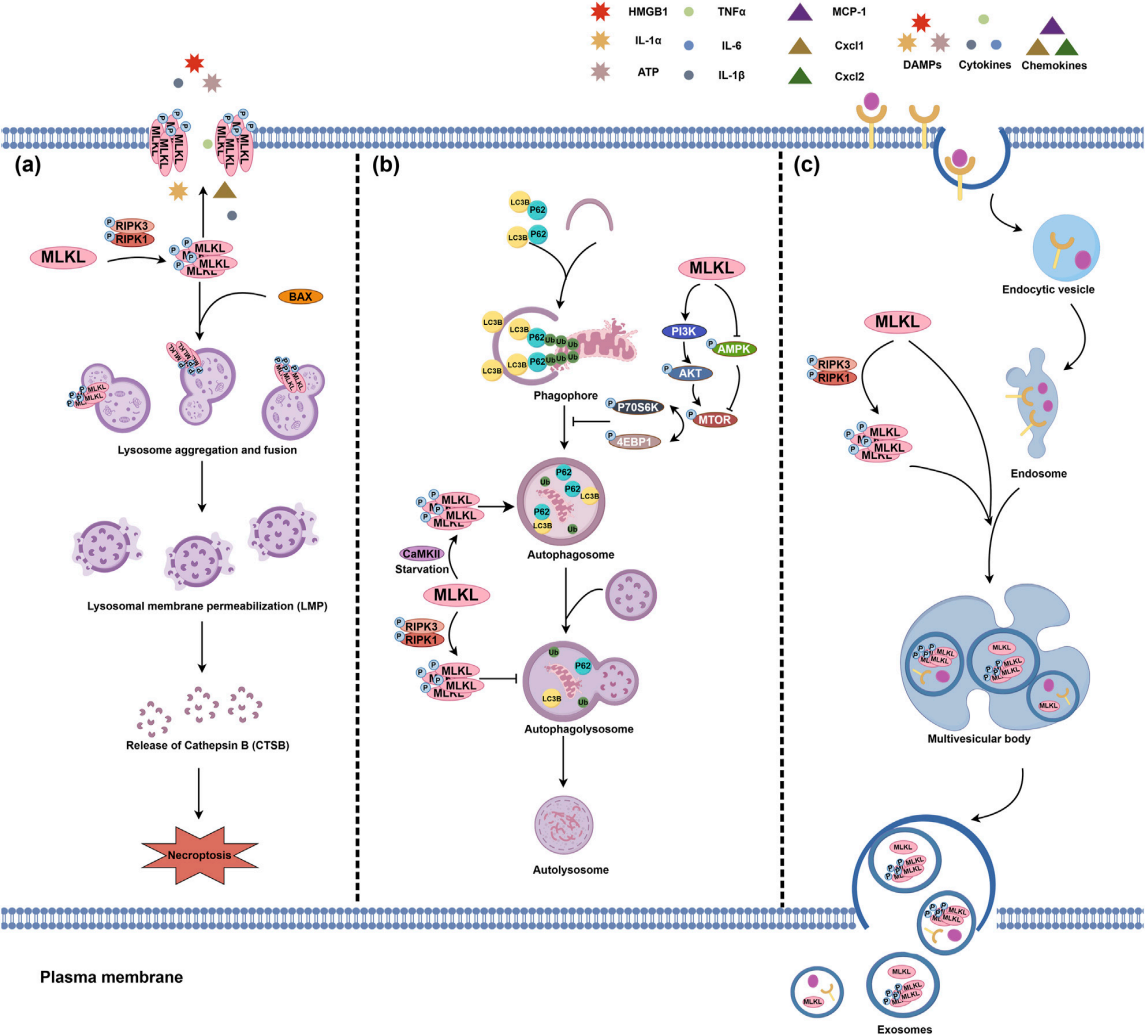
The pathophysiological actions of MLKL in the lysosome and endosome. **(A)** MLKL translocates to the lysosomal membrane, triggering lysosome aggregation and fusion and ultimately leading to lysosomal membrane permeabilization (LMP). The interaction between p-MLKL and BAX is also a LMP inducer. LMP may result in the release of lysosomal contents, such as CTSB for cleaving pro-survival proteins and consequently trigger necroptosis. **(B)** MLKL also plays a pivotal role in lysosomal autophagy. MLKL can promote the phosphorylation of mTOR, p70S6K, and 4EBP1 by enhancing the PI3K/AKT signaling pathway and inhibiting AMPK phosphorylation, which may impair the formation of autophagosomes. In contrast to canonical RIPK3 phosphorylation, CaMKII-mediated non-canonical MLKL phosphorylation promotes autolysosomal formation. **(C)** MLKL is also involved in endosomal transport. Multiple receptors on the plasma membrane can enter the intracellular region via endocytosis and are delivered to the endosomes via endocytic vesicles. MLKL, on the one hand, can strengthen the transport of endosomes to multivesicular bodies, thereby expediting the degradation of intracellular ligands and receptors and the release of exosomes. On the other hand, activated MLKL can further enhance the efficacy of this endosomal transport and lead to the release of p-MLKL from extracellular vesicles, thereby preventing the aggregation of p-MLKL on the plasma membrane and the severity of necroptosis.

Autophagy is another major function of the lysosome, which can also be impacted by MLKL (Zhang et al., 2021; Dong et al., 2023; Frank et al., 2019; Guo et al., 2019; Zhan et al., 2022). The mammalian target of rapamycin (mTOR) signaling pathway is a critical regulator of autolysosome formation (Zhu et al., 2019; Xu et al., 2021). MLKL can activate the PI3K/AKT signaling to promote mTOR phosphorylation and inhibit AMPK phosphorylation, which then increases p70S6K and 4EBP1 phosphorylation levels. This ultimately leads to the impair of autolysosome formation and autophagy process (Frank et al., 2019; Guo et al., 2019; Sui et al., 2023). However, it is paradoxical about the role of MLKL in autophagy regulation in tumor cells, such as mouse Neuro-2a and L929 cells, and human HEK293 and HT29 cells (Zhan et al., 2022). During cell starvation, CaMKII, a key downstream effector of cytosolic Ca^2+^ signaling, is activated; CaMKII activation, rather than RIPK3, promotes MLKL phosphorylation and oligomerization, facilitating the separation of autophagosome membranes and fusion with the lysosome, thus enhancing autophagic flux (Zhan et al., 2022; Xu et al., 2019; Xu et al., 2020). However, whether the regulations of MLKL on autophagy, induction or inhibition, is good or bad for the consequence of cells may be correlated with the cellular status, which needs further exploration (Figure 2).

### 3.3 MLKL in the endosome

The endosome is a core hub for vesicle transport, and thus plays a pivotal role in substance transport and sorting, homeostasis maintenance, and signal transduction (Wang et al., 2022; Lu et al., 2022a). According to the features and functions during endocytosis, endosomes can be classified into early, late, and recycling endosomes (Scott et al., 2014). MLKL can translocate to early and late endosomes, accelerate the trafficking of ligand/receptor complexes, such as EGF/EGFR, and death-inducing signals TNF/TNFRI and TRAIL/TRAIL-death receptors 5 (DR5), from early to late endosomes, leading to their degradation and promoting extracellular vesicles generation (Wu et al., 2023; Yoon et al., 2017; Park et al., 2020). RIPK3 deficiency does not affect this function of MLKL, however, the phosphorylation of MLKL by RIPK3 can promote its regulatory function on the endosome transport and the release of more extracellular vesicles containing p-MLKL, which in part helps with reducing plasma membrane permeabilization and cell death (Yoon et al., 2017).

MLKL-mediated endosomal transport also participates in the homeostatic regulation of lipids. This process involves the interaction between MLKL and the endosomal sorting complexes required for transport (ESCRT) machinery, where MLKL-driven ESCRT promotes the trafficking of modified lipoproteins and facilitates the transition from early endosomes to multivesicular bodies in late endosomes by maintaining the balance of phosphatidylinositol phosphates (Rasheed et al., 2020). The regulations of MLKL on the endosome homeostasis are summarized in Figure 2.

### 3.4 MLKL in the endoplasmic reticulum

The endoplasmic reticulum is the primary site for the synthesis, processing, packaging, and transport of secreted and transmembrane proteins (Chen and Cubillos-Ruiz, 2021). On the one hand, the translocation of MLKL oligomer to the endoplasmic reticulum membrane causes membrane damage (Liang et al., 2021). On the other hand, MLKL can directly induce the activation of endoplasmic reticulum stress (ERS)-related pathways (Liang et al., 2021). ERS can activate three classical endoplasmic reticulum-localized transmembrane sensors of the unfolded protein response (UPR), including activating transcription factor 6 (ATF6), inositol-requiring enzyme 1α (IRE1α) and PRKR-like endoplasmic reticulum kinase (PERK) (Haze et al., 1999; Yoshida et al., 2001; Harding et al., 1999). Upon ERS, the accumulation of unfolded proteins sequestrates the chaperone GRP78 (BiP) from UPR sensors for activating IRE1, PERK, and ATF6 (Hetz et al., 2020; Yuan et al., 2021; Xia et al., 2023; Huang C. et al., 2022). However, oligomerized MLKL can rapidly directly phosphorylate PERK and IRE1α without disrupting their interaction with GRP78, consequently trigger a non-canonical ERS response (Liang et al., 2021).

**FIGURE 3:**
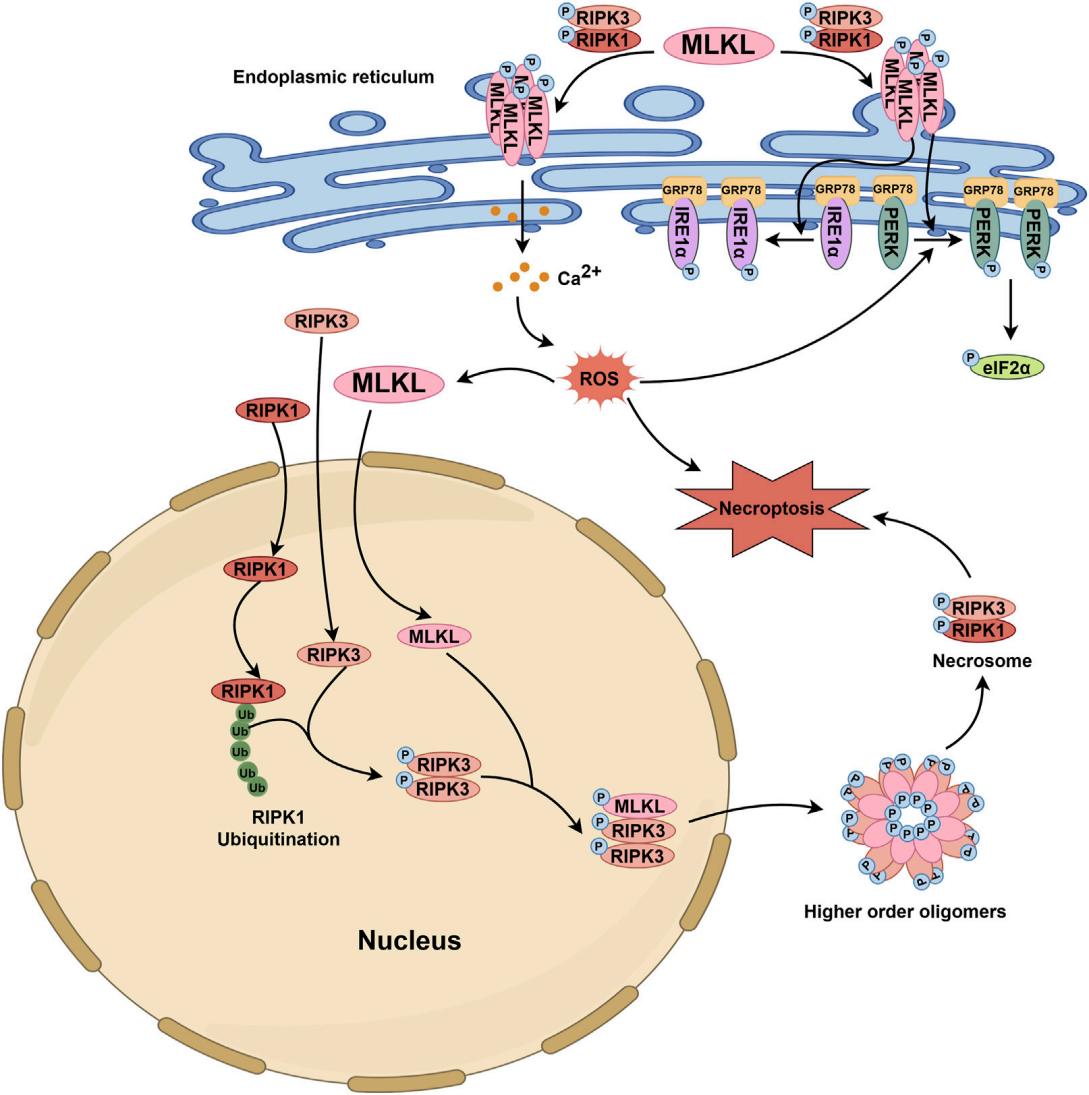
MLKL-related pathophysiological events in the endoplasmic reticulum and nucleus. Upon stimulation, MLKL is phosphorylated through the RIPK1/RIPK3 axis. Subsequently, phosphorylated MLKL translocates to the endoplasmic reticulum membrane, inducing structural disruption and promoting the release of Ca^2+^ from the endoplasmic reticulum into the cytoplasm. This leads to an increase in cytoplasmic ROS levels. Additionally, p-MLKL can activate UPR sensors such as IRE1α and PERK, which bind to GRP78, thereby initiating their phosphorylation and triggering an unconventional ERS response. The elevated ROS in the cytoplasm further enhances the activation of the PERK-eIF2α pathway. During necroptosis, RIPK3 and MLKL function as nuclear transport proteins, shuttling continuously between the nucleus and cytoplasm, while RIPK1 remains localized exclusively in either the nucleus or cytoplasm. Ubiquitination of nuclear RIPK1 provides a platform for the activation of nuclear RIPK3. Phosphorylated RIPK3 then interacts with MLKL, leading to MLKL phosphorylation. Both phosphorylated RIPK3 and MLKL then exit the nucleus together to form high-order oligomers, which further promote the formation of the cytosolic necrosome and ultimately lead to cell death. The activation of the PERK-eIF2α pathway and the increase in cytoplasmic ROS levels also facilitate the nuclear entry of MLKL.

The endoplasmic reticulum is also the intracellular Ca^2+^ pool. Activated MLKL accumulates on the endoplasmic reticulum and benefits the influx of cation-Ca^2+^, leading to calcium overload in the endoplasmic reticulum and ERS (Gong et al., 2017). However, as we mentioned earlier, MLKL also regulates Mg^2+^dynamics by forming cation channels and shuttling Mg^2+^ between the endoplasmic reticulum and the mitochondrion (Jiang et al., 2023). In the endoplasmic reticulum, the MLKL channel enables Mg^2+^ across and to be released in order to keep the endoplasmic reticulum in homeostasis and away from swelling (Figure 3).

These findings, although reveals the modulation of MLKL on the homeostasis of the endoplasmic reticulum, are far away from uncovering the molecular basis of MLKL, which means that more in-depth studies are needed for putting forward the application of MLKL in resolving endoplasmic reticulum-related issues.

### 3.5 MLKL in the nucleus

The nucleus stores genetic material and controls cellular genetic and metabolic activities. MLKL is a nucleo-cytoplasmic shuttling protein that can translocate between the cytoplasm and the nucleus before heading to the plasma membrane for rupture. The phosphorylation and conformational switch of MLKL to expose the bipartite nuclear localization signal (NLS, amino acids 224–256) in its C-terminal region is critical for its nuclear transport, especially the export but not the import (Yoon et al., 2016). More specifically, MLKL enters the nucleus via the Ran-GTP/importin-dependent pathway and is phosphorylated by activated RIPK3 within the nucleus; then, p-MLKL and RIPK3 co-transport to the cytoplasm, where they oligomerize into a necrosome and MLKL forms higher-order oligomers that are detrimental to the plasma membrane (Weber et al., 2018). In addition, nuclear MLKL itself is adequate for triggering necroptosis independent of RIPK1/RIPK3, according to the observation that necroptosis could be induced by single expression of MLKL-L280/283/284A (a mutant form with a nuclear export signal sequence of MLKL) and inhibited by the nuclear import inhibitor GppNHp but not RIPK1/RIPK3 inhibition (Ino et al., 2023). MLKL inhibitors that can block its execution of necroptosis do not affect its nuclear translocation. The nuclear MLKL is also involved in the apoptotic signal, where it responds to ROS and interacts with the PERK–eIF2α pathway for apoptosis initiation (Cao et al., 2018).

MLKL translocation to the nucleus has been observed in various mouse and human cell lines under the stimulation of different pro-death signals (Figure 3). However, further researches are needed to specify whether there is a difference in the mechanism for MLKL nuclear transport between human and mouse, in order to propel the clinical translation of nuclei-regulatory formulations targeting MLKL.

## 4 MLKL as a potential therapeutic target for diseases

**FIGURE 4:**
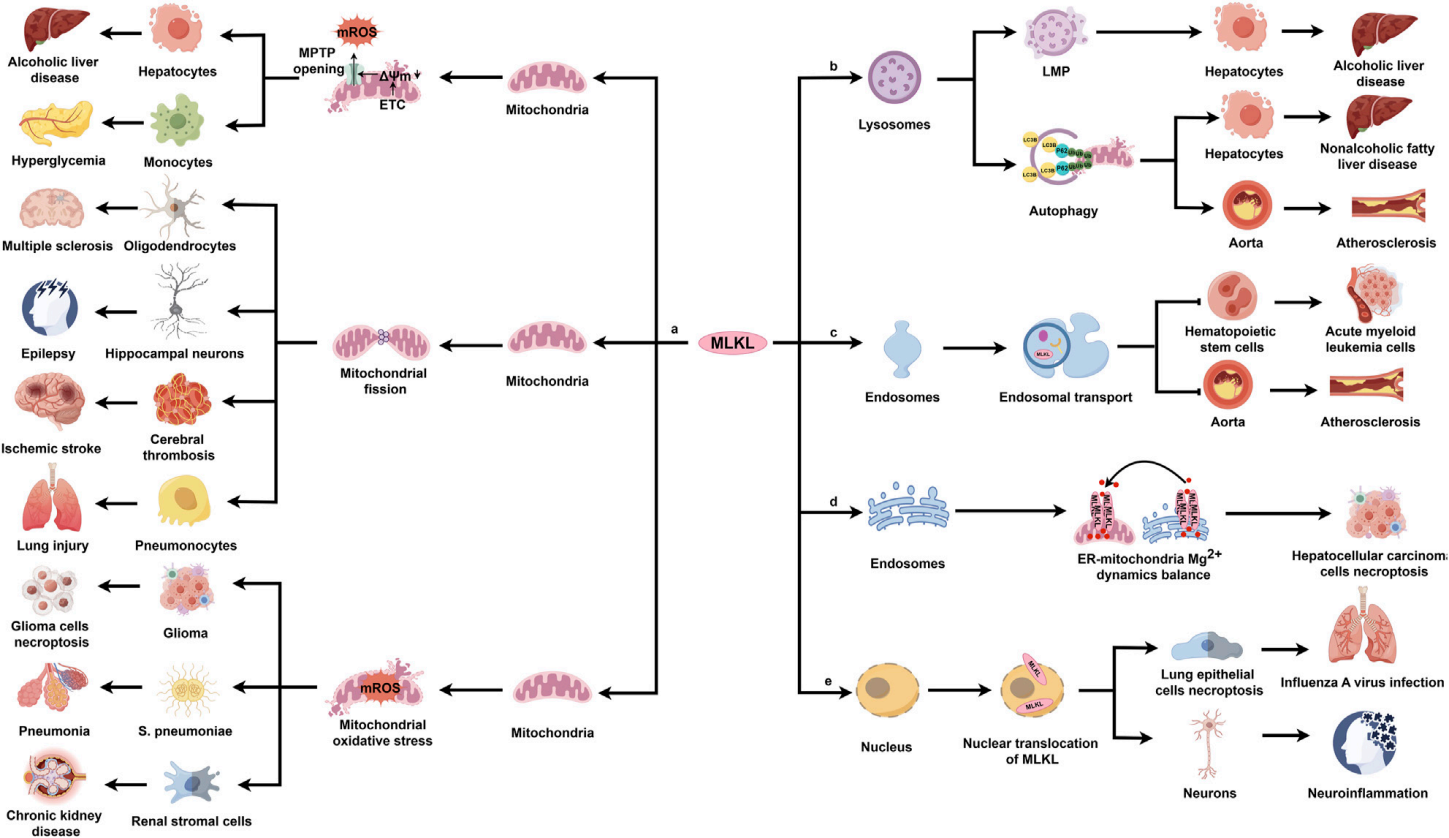
The pathogenetic actions and mechanisms of MLKL in various diseases. MLKL is ubiquitously expressed in multiple organs, including the nervous system, cardiovascular system, respiratory system, digestive system, and urinary system. In response to various pathological stimuli, MLKL translocates to different organelles, where it performs distinct regulatory functions and results in disease development.

The translocation of MLKL to diverse organelles have been validated in cell researches, and more importantly, this translocation is of significance to the cell biology. However, the *in vivo* role of MLKL that targets specific organelles in diseases is still unclear. In the following section, we are going to discuss how MLKL in specific organelles contributes to the pathogenesis of various diseases (Figure 4).

### 4.1 MLKL-mediated mitochondrial dysfunction in diseases

MLKL triggers mitochondrial dysfunction through necroptosis-dependent and -independent mechanisms. Necroptosis-activated MLKL can directly disrupt the mitochondrial membrane. For example, ethanol exposure mobilizes p-MLKL to the outer mitochondrial membrane in hepatocytes during alcoholic liver disease, contributing to the opening of MPTP and the release of damage-associated molecular patterns; pharmacological inhibition of MLKL with tetramethylpyrazine reverses this pathology (Zhou et al., 2022). Consistently, another study found that ROS promotes MLKL trafficking to the mitochondrion under hyperglycemic conditions, leading to the formation of high molecular weight MLKL oligomers in the mitochondrial membrane and the opening of MPTP (Deragon et al., 2023). Alternatively, during necroptosis, MLKL activates multiple downstream factors to indirectly damage the mitochondrial membrane. Several studies have highlighted the critical role of MLKL/PGAM5/Drp1 signaling pathway in damaging the mitochondrion through necroptosis-dependent manner in disease models (Luo et al., 2017; Abd El-Aal et al., 2022; Hu et al., 2022; Ling et al., 2022; Lu et al., 2022b). One example is multiple sclerosis, a chronic autoimmune disease affecting the central nervous system, characterized by oligodendrocyte loss, demyelination, inflammation, and neuronal degeneration (Oh et al., 2018). Animal models showed that MLKL phosphorylation via the RIPK1/RIPK3 axis activates PGAM5 and induces Drp1 translocation to the mitochondrion, increasing mitochondrial fragmentation, which correlates with the axonal damage in multiple sclerosis lesions and leads to oligodendrocyte death (Luo et al., 2017). Similarly, the MLKL/PGAM5/Drp1 pathway can be activated in epilepsy, a neurological condition affecting thoughts and behaviors. A recent study found increased MLKL protein expression in neurons of Wistar rats after 25 days of pentylenetetrazole (PTZ) exposure. Pharmacological inhibition of MLKL by morin significantly improves epilepsy, restoring cognitive decline and memory impairment and the preserving hippocampal neurons. Mechanistically, morin inhibits MLKL expression and its downstream cascade, reducing mitochondrial fragmentation and protecting hippocampal neurons (Abd El-Aal et al., 2022). *Panax notoginseng* saponins (PNS) and acteoside (AC) play similar roles in treating ischemic stroke (Hu et al., 2022) and RSV-induced lung injury (Ling et al., 2022), respectively. CypD, another downstream factor in the MLKL/PGAM5 pathway, is involved in various diseases (Zhang S. L. et al., 2019). For example, prolactinoma, a common benign tumor, accounts for about 40%–60% of pituitary adenomas (Saleem et al., 2018). Bromocriptine triggers necroptosis in prolactinoma cells by enhancing mitochondrial permeability and inducing mitochondrial swelling via the MLKL/PGAM5/CypD pathway (Zhang S. L. et al., 2019). Additionally, mitochondrial ROS (mROS), a key marker of mitochondrial dysfunction, significantly impact numerous disorders (Srivastava et al., 2022; Huang et al., 2021; Ding et al., 2019). During an infection with *Scutellaria pneumoniae*, MLKL interacts with RIPK1, RIPK3, and MCU (mitochondrial calcium uniporter) to induce mitochondrial calcium uptake and the production of mROS (Huang et al., 2021). MLKL knockout mice show reduced resistance to lung injury, bacterial load, and inflammation compared to WT mice after intranasal instillation with *S. pneumoniae* (Huang et al., 2021). These findings indicate that MLKL’s association with the mitochondrial membrane is crucial for the response to *S. pneumoniae* (Huang et al., 2021). Anjali et al. recently discovered that necroptosis activation enhances MLKL’s translocation to mitochondria, leading to increased mROS production in kidneys of mice with oxalate-induced chronic kidney disease (CKD). This mROS then activates CaMKII in the mitochondria, promoting the development of intrarenal extracellular matrix, a common feature of CKD (Srivastava et al., 2022). Similarly, these findings can be also explored in glioma (Ding et al., 2019). A key aspect of glioma cell necroptosis is the persistent accumulation of mROS throughout the disease. Shikonin, a naphthoquinone derived from *Lithospermum erythrorhizon*, has shown significant therapeutic effects on glioma (Lu et al., 2017). Mechanistically, shikonin activates MLKL, which targets mitochondria and increases mitochondrial superoxide production. This enhances γ-H2AX synthesis via elevated mROS, leading to chromatinolysis and ultimately cell death (Ding et al., 2019).

Additionally, MLKL modulates mitochondrial independently of necroptosis, contributing to various diseases. For instance, in liver ischemia and reperfusion (IR) injury (Xu J. et al., 2023), a common issue after surgeries like liver transplantation (Peralta et al., 2013), MLKL knockout mitigates IR-induced liver damage and macrophage pro-inflammatory responses compared to control mice (Xu J. et al., 2023). In MLKL-knockout intrahepatic macrophages, PINK1-mediated mitophagy is enhanced, and hepatocyte oxidative DNA damage is reduced, suggesting that MLKL suppresses mitophagy and exacerbates oxidative DNA damage (Xu J. et al., 2023).

Currently, MLKL’s regulation of mitochondrial function in diseases is primarily limited to necroptosis with its non-necroptotic role in mitochondria receiving little attention. Exploring these non-necroptotic functions may offer a promising direction for future disease research.

### 4.2 MLKL-mediated lysosomal dysfunction in diseases

Lysosomes, essential membrane-enclosed organelles, require an intact cell membrane for normal functions. However, MLKL can trigger LMP in pathological conditions, influencing autophagy or cell death (Liu S. et al., 2024). For example, palmitic acid (PA)-induced lipotoxicity can cause LMP in alcoholic liver disease (ALD), characterized by increased galectin-3 puncta formation, a key indicator of LMP in live cells (Dong et al., 2023). Activated BAX upregulates phosphorylated MLKL, which then translocates to the lysosomal lipid bilayer, inducing LMP. This leads to lysosomal permeabilization and release of lysosomal contents into the cytosol, resulting in hepatocyte death (Dong et al., 2023). Notably, silencing MLKL restores LAMP2 levels, a lysosomal membrane protein involved in autophagosome-lysosome fusion, suggesting that MLKL may inhibit autophagy flux and contribute to autophagic cell death in ALD (Dong et al., 2023). Another study on non-alcoholic fatty liver disease (NAFLD) further supports the link between MLKL and suppressed autophagic flux in hepatocytes (Wu et al., 2020). MLKL gene expression is upregulated in murine livers after a 12-week Western diet (FFC diet) and in AML12 and primary mouse hepatocytes exposed to PA for 16 h (Wu et al., 2020). MLKL increases plasma aminotransferase levels and hepatic triglyceride concentrations in mice fed a FFC diet, which inhibits lipophagy (autophagic degradation of lipid droplets). MLKL knockout can protect mice from FCC-induxed liver damage (Wu et al., 2020). A possible mechanism is that MLKL translocates to autophagosomes, blocking their fusion with lysosomes and leading to the accumulation of p62 and LC3-II (Wu et al., 2020).

Additionally, two studies highlight the critical role of MLKL signaling pathway in regulating autophagy during atherosclerosis onset (Guo et al., 2019; Ma et al., 2022). In one study, MLKL is upregulated in atherosclerotic plaques from patients with carotid disease and in apoE^−/−^ mice on a high-fat diet. Pharmacological inhibition of MLKL with calycosin significantly improves atherosclerosis, reducing necrotic core area and vascular inflammation, as well as foam cell formation (Ma et al., 2022). Mechanistically, calycosin-induced Krüppel-like Factor 2 (KLF2) negatively regulates MLKL, enhancing autophagy and cholesterol efflux to reduce lipid accumulation, inflammation, and apoptosis in macrophages (Ma et al., 2022). In another study, oxidized low-density lipoprotein (OX-LDL) worsens atherosclerosis by inhibiting autophagy through MLKL. This may involve MLKL induced by LncRNA-FA2H-2, which impairs autophagy via AMPK-mTOR axis, increasing pro-inflammatory factors and DAMPs release (Guo et al., 2019).

In summary, ample evidence indicates that inhibiting MLKL-induced LMP or autophagy can improve various diseases. Maintaining or enhancing lysosomal function by reducing MLKL expression with effective drugs may be a promising treatment strategy.

### 4.3 MLKL-mediated endosomal dysfunction in diseases

As previously noted, MLKL within endosomes regulates the trafficking of internalized proteins, aiding in receptor and ligand degradation, modulating signaling, and promoting extracellular vesicle formation (Yoon et al., 2017). Notably, MLKL’s role in endosomal trafficking can occur independently of RIPK3, unlike its role in necroptosis. Several studies indicate that MLKL-mediated endosomal transport significantly impacts various diseases (Park et al., 2020; Rasheed et al., 2020; Wang et al., 2021). For example, MLKL acts as a tumor suppressor by increasing endosomal trafficking of granulocyte colony-stimulating factor (G-CSF) in acute myeloid leukemia (AML) cells (Wang et al., 2021). This is significant because the increased release of G-CSF promotes the proliferation and differentiation of myeloid progenitor cells, which is a key pathological feature of AML (Skokowa et al., 2009; Begley et al., 1987). It was found that MLKL loss prevents G-CSF from efficiently entering the endosomal compartment, as evidenced by a significant reduction in the co-localization of G-CSF with the early endosomal marker EEA1 in MLKL knockout cells compared to WT cells, despite similar intracellular EEA1 levels (Wang et al., 2021). In HeLa, H2009, and MDA-MB231 cancer cells, MLKL enhances cancer cell death by increasing endocytosis and endosomal trafficking of TRAIL-DR5 apoptotic signals compared to control cells (Park et al., 2020). Similarly, MLKL-mediated endosomal trafficking is implicated in the pathogenesis of atherogenesis (Rasheed et al., 2020). MLKL is found in late endosomes and multivesicular bodies in peritoneal macrophages after exposure to atherogenic lipoproteins (Rasheed et al., 2020). However, MLKL knockdown increases lipid localization in multivesicular bodies, suggesting impaired lipid trafficking and accumulation in macrophages (Rasheed et al., 2020). Additionally, PIP3 staining also increases in macrophages treated with oxidized low-density lipoprotein (LDL) after MLKL knockdown, indicating disrupted endocytic pathway maturation (Rasheed et al., 2020). This phenomenon may involve MLKL promoting the trafficking of modified lipoproteins by interacting with the ESCRT and facilitating the transition from early endosomes to multivesicular bodies by maintaining PIP balance (Rasheed et al., 2020). The data suggest that MLKL inhibits lipid accumulation in macrophages by facilitating endosome maturation and intracellular trafficking of modified lipoproteins in atherogenic conditions (Liang et al., 2023).

### 4.4 MLKL-mediated endoplasmic reticulum dysfunction in diseases

ERS contributes to cell death in various cancers, including hepatocellular carcinoma (HCC) (Lin et al., 2019). MLKL expression is significantly higher in HCC tissues compared to adjacent non-cancerous tissues, correlating with patient survival (Jiang et al., 2023). Knocking out MLKL induces parthanatos in Hepa one to six cells, a type of cell death linked to oxidative stress due to impaired UPR signaling activation caused by MLKL deficiency (Jiang et al., 2023). Notably, MLKL’s regulation of ERS in HCC suggests a mechanism where MLKL on the membrane forms cation channels, permeability permeable to Mg^2+^. Depletion of MLKL in the endoplasmic reticulum membrane impairs Mg^2+^ release, leading to endoplasmic reticulum swelling and inhibited UPR signaling (Jiang et al., 2023). Current understanding of MLKL’s role in endoplasmic reticulum regulation is limited to ERS. Further investigation into the proteins or metabolites in the endoplasmic reticulum involved in MLKL activation and the mechanisms of MLKL-mediated endoplasmic reticulum dysfunction is of great interest.

### 4.5 MLKL-mediated nuclear dysfunction in diseases

To date, research on MLKL’s nuclear role has primarily focused on its involvement in necroptosis, especially in viral-induced pathologies (Zhang T. et al., 2020; Prasad Panda et al., 2023). Influenza A virus (IAV), an enveloped virus, can cause seasonal epidemics and pandemics (Zhao et al., 2017). MLKL knockout mice exposed to lethal doses of IAV show reduced nuclear disruption in lung epithelia, decreased neutrophil recruitment, and improved survival compared to WT mice (Zhang T. et al., 2020). Mechanistically, IAV produces Z-RNAs to activate the host protein ZBP1 in nucleus, which stimulates RIPK3 to phosphorylate MLKL. MLKL then ruptures the nuclear membrane, releasing DNA into the cytoplasm and attracting neutrophils (Zhang T. et al., 2020). Additionally, in neuroinflammatory diseases, MLKL translocates to the nucleus, initiating NLRP3 inflammasome transcription (Prasad Panda et al., 2023). MLKL-induced NLRP3 activity leads to caspasese-1 cleavage and IL-1 activation, causing neuroinflammation (Prasad Panda et al., 2023; Cheng J. et al., 2021; Du et al., 2019), a complex response involving immune cell activation and migration (Bonora et al., 2022). This suggests that nuclear MLKL may drive inflammation in these disorders. Understanding MLKL’s nuclear function in disease model is crucial for developing new treatment strategies.

## 5 MLKL inhibitors from distinct sources

### 5.1 Natural products

Natural products belong to a category of biologically active compounds synthesized by living organisms, including plants, animals, insects, marine organism, and microbes (Zhu et al., 2022; Liu Q. et al., 2024). Most natural products share a common advantage of diverse sources, which makes them a rich source for discovering MLKL-related drugs. The validated compounds are classified into three types according to their different mechanisms: compounds that can limit MLKL expression, compounds that inhibit MLKL phosphorylation, and compounds that targets phosphorylated MLKL oligomerization.

#### 5.1.1 Natural products that limit MLKL expression

**FIGURE 5:**
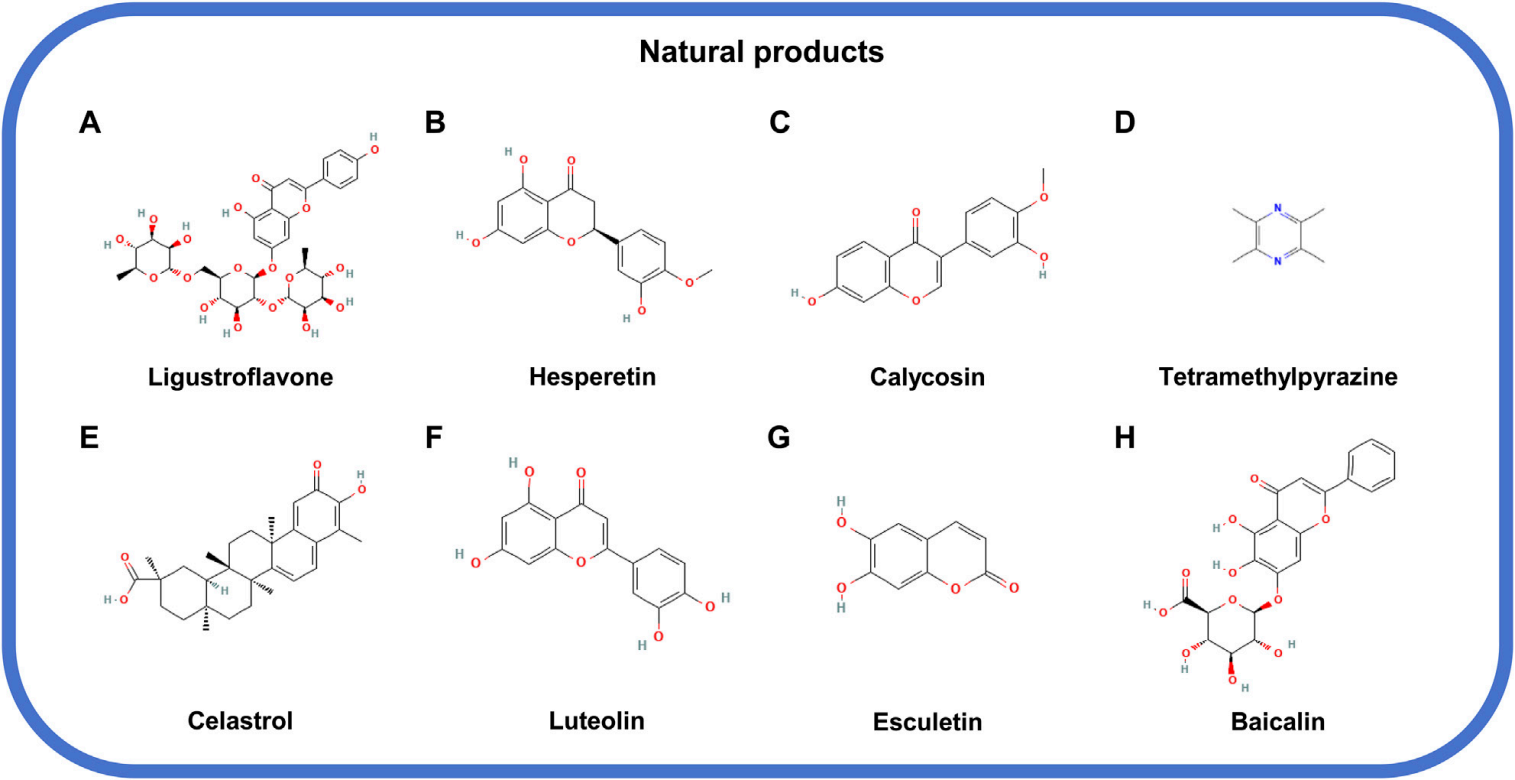
The structural formula of natural products inhibiting MLKL. **(A)** Ligustroflavone. **(B)** Hesperetin. **(C)** Calycosin. **(D)** Tetramethylpyrazine. **(E)** Celastrol. **(F)** Luteolin. **(G)** Esculetin. **(H)** Baicalin.

Ligustroflavone, hesperetin, and calycosin belong to the category of natural compounds that target MLKL expression. Ligustroflavone (Figure 5A), extracted from *Ligustrum Lucidum*, has a strong anti-inflammatory activity and therapeutical effect on diseases such as ischemic stroke, which was found to be related with its suppression on MLKL expression (Zhang Y. Y. et al., 2019; Cruz et al., 2018). Consistently, the anti-inflammatory effect of hesperetin (Figure 5B), a flavanone existed in citrus fruits, on colitis, is also attributed by its suppression on MLKL expression, necroptosis blockade, and immune responses (Shirzad et al., 2017; Alu’datt et al., 2017; Zhang J. et al., 2020). The interaction between KLF2 and MLKL is critical for the stable expression of MLKL, which when disrupted, by calycosin (Figure 5C) from *Radix Astragali*, for example, could directly cause MLKL decline (Ma et al., 2022; Wu et al., 2006).

#### 5.1.2 Natural products that suppress MLKL phosphorylation

The natural compounds targeting MLKL phosphorylation include tetramethylpyrazine, celastrol, luteolin, and esculetin. Tetramethylpyrazine (Figure 5D) derived from *Ligusticum Wallichii* and celastrol (Figure 5E), isolated from *Tripterygium Wilfordii* could inhibit the phosphorylation of MLKL to halt cell necrosis and tissue injury (Zhou et al., 2022; Liang et al., 2023). On the one hand, the direct binding to MLKL by compounds, such as luteolin (Figure 5F), a flavonoid from *Rhizoma Drynariae*, could prevent MLKL from being phosphorylated and functioning in necrosis (Xu X. et al., 2023). On the other hand, the compound like esculetin [6,7-Dihydroxycoumarin (DHC); Figure 5G] could block the ATP-binding site of MLKL and reduce MLKL phosphorylation (Prajapati et al., 2021).

#### 5.1.3 Natural products that target phosphorylated MLKL oligomerization

Baicalin (Figure 5H), extracted from the root of *Scutellaria baicalensis* Georgi, is an antagonist of MLKL oligomers. Baicalin has no effects on the phosphorylation of RIPK1, RIPK3, and MLKL but significantly lessen the oligomerization of MLKL, which also can impair the occurrence of necroptosis and pathological changes such as inflammation (Huang Y. T. et al., 2022; Hu et al., 2021).

### 5.2 Chemical compounds

#### 5.2.1 Small-molecule chemical compounds

Multiple small-molecule chemical compounds attract our attention owing to their efficiency in disrupting MLKL polymerization and membrane translocation. Unlike macromolecules, small molecules can easily penetrate the plasma membrane and interact with target proteins due to their small size. According to the structural features and action patterns, the small-molecule MLKL inhibitors are divided into covalent (Liao et al., 2014; Yan et al., 2017; Cui et al., 2022; Tang and Zhuang, 2024) and non-covalent inhibitors (Hildebrand et al., 2014; Pierotti et al., 2020; Rubbelke et al., 2021).

##### 5.2.1.1 Covalent inhibitors

**FIGURE 6:**
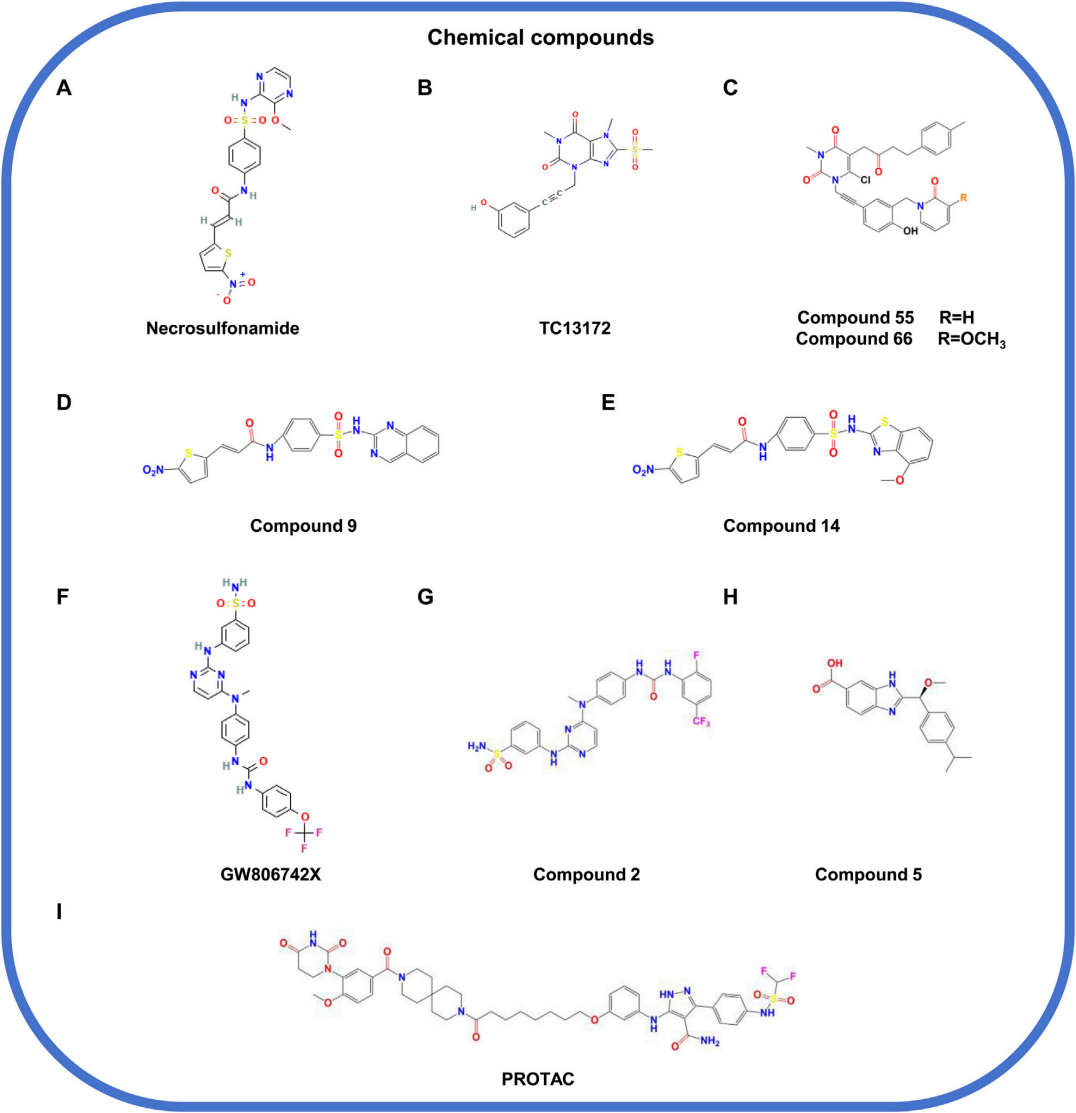
The structural formula of chemical compounds targeting MLKL. **(A)** Necrosulfonamide. **(B)** TC13172. **(C)** Compound 55/66. **(D)** Compound 9. **(E)** Compound 14. **(F)** GW806742X. **(G)** Compound 2. **(H)** Compound 5. **(I)** PROTAC.

Necrosulfonamide (NSA; Figure 6A), an early irreversible covalent inhibitor of MLKL, is widely used in preclinical researches (Liao et al., 2014). It can specifically target C86 in the 4HBD of human MLKL, partially blocking its oligomerization and membrane translocation (Rubbelke et al., 2020). However, the limited potency and narrow structure-activity relationship (SAR) limit its development in drug exploration. TC13172 (Figure 6B), another covalent inhibitor, shares a similar mechanism with NSA but is more efficient in inhibiting MLKL oligomerization (Yan et al., 2017). Based on the structure of TC13172, novel MLKL inhibitors with uracil nuclei (compound 55 and compound 66; Figure 6C) were synthesized, which show stronger capacity of inhibiting MLKL membrane translocation but weaker capacity of inhibiting its oligomerization (Cui et al., 2022). However, these inhibitors are ineffective in murine cells due to the replacement of cysteine with tryptophan at C86 of MLKL (Sun et al., 2012). Compounds nine and compound 14 (Figures 6D, E), derived from NSA with fused heterocycles, have powerful anti-necroptotic activity by inhibiting MLKL oligomerization and translocation (Tang and Zhuang, 2024).

Collectively, covalent inhibitors function based on their binding to MLKL and thereby inhibition on MLKL oligomerization and membrane translocation but not phosphorylation. However, the efficacy of these inhibitors is still needed to be validated *in vivo* and far away from being moved into clinical researches.

##### 5.2.1.2 Non-covalent inhibitors

Compound 1 (GW806742X) (Figure 6F) is a non-covalent inhibitor that targets the PsKD of MLKL by directly interacting with its nucleotide-binding site, thus inhibiting the conformational changes of MLKL and the occurrence of necroptosis (Hildebrand et al., 2014). Its derivative, compound 2 (Figure 6G), has a stronger activity and can bind to the PsKD or full-length of MLKL, forming hydrogen bonds with residues including E250, G349, and C286 (Pierotti et al., 2020). Both compounds can significantly inhibit necroptosis but lack specificity, affecting other kinases such as RIPK1, RIPK3, and VEGFR2 (Hildebrand et al., 2014; Pierotti et al., 2020). Another study validated the compound 5 (Figure 6H) that can target the 4HBD of MLKL (Rubbelke et al., 2021). However, the low membrane permeability and negative liposome leakage of compound 5 limits its *in vivo* application, where optimization is needed for propelling its development as a MLKL inhibitor candidate.

Although the non-covalent inhibitors of MLKL show good performance in suppressing necroptosis, these inhibitors lack enough specificity and may affect other signals. Thus, further researches should focus on enhancing the preciosity and safety of these inhibitors.

#### 5.2.2 Proteolysis-targeting chimera (PROTAC)

PROTAC is a technology utilizing the ubiquitin-proteasome system (UPS) to degrade specific proteins. In detail, the technique works through a small molecule chimera that combines the target protein of interest to degrade with the E3 ubiquitin ligase; this binding causes the target protein to be labeled (ubiquitination) and subsequently degraded by the proteasome within the cell (Nalawansha and Crews, 2020; Hu M. et al., 2020; Chen et al., 2022; Yao et al., 2024). Unlike traditional small-molecule inhibitors, PROTACs are featured by the advantages such as catalytic specificity, low dosage, and longer-lasting effects (Mao et al., 2022). A recent study designed a PROTAC (Figure 6I) based on a high-affinity pyrazolformamide MLKL ligand, which can specifically target MLKL for degradation and inhibit cell death (Rathje et al., 2023).

Despite that PROTACs are well performed in the specificity, challenges still exist due to their large molecular weight and poor bioavailability. More researches are still required for optimizing this kind of MLKL inhibitors and accelerate their application.

### 5.3 Drugs used in clinical practice

**FIGURE 7:**
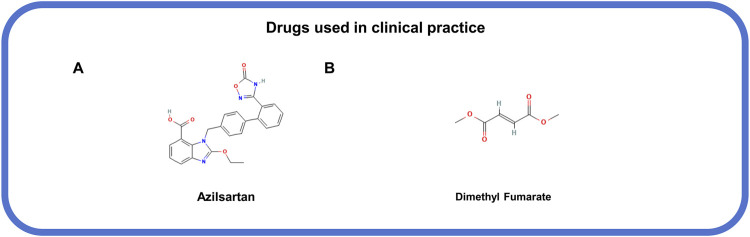
The structural formula of MLKL-related drugs used in clinical practice. **(A)** Azilsartan. **(B)** Dimethyl fumarate.

Several drugs that used in clinical have been confirmed to action in association with the inhibition of MLKL phosphorylation and oligomerization (Marunouchi et al., 2024; Shi et al., 2023). Azilsartan (Figure 7A), an angiotensin II receptor antagonist, and trandolapril, an angiotensin-converting enzyme inhibitor (Marunouchi et al., 2024), both show inhibitory effects on MLKL phosphorylation during heart failure following angiotensin II-induced myocardial infarction (Marunouchi et al., 2024). Dimethyl fumarate (Figure 7B), an anti-inflammatory drug approved for relapsing-remitting multiple sclerosis in the United States and the Europe (Shi et al., 2023), can indirectly inhibit MLKL activation and necroptosis by suppressing the ubiquitination of RIPK1 and RIPK3 (Shi et al., 2023). However, the indications for these drugs should be taken into careful consideration based on specific conditions, for that these drugs can typically target other signals and cause unintentional consequences.

## 6 Conclusion

In this review, we have elaborated the structural feature of MLKL that is the basis for its function and crucial to drug invention. Further, we have summarized the regulation of MLKL on various cellular organelles, such as the mitochondrion, lysosome, endosome, endoplasmic reticulum, and nucleus. Also, we have drawn the interaction network of components involved in the actions of MLKL across these diverse organelles. Based on its regulatory functions on the organelles, MLKL is involved in or even contributes to the occurrence and development of multiple conditions, including cancer, neurological, metabolic, cardiovascular, and respiratory diseases. The preclinical interventions on MLKL may improve the disease outcome without bring obvious adverse effects, which validates that MLKL may be a promising therapeutic target. Moreover, we have listed several compounds with inhibitory effects on MLKL, which are promising drug candidates for disease therapy.

### 6.1 Future directions and challenges

The regulatory roles of MLKL in some organelles have been preliminarily revealed, the detailed mechanisms still need further exploration, especially how MLKL is involved in the interaction among these organelles and whether these interactions in reverse act on MLKL functions. Besides, the roles of MLKL in other organelles, such as golgi, remain unknown.

In some circumstances, MLKL can be released to the extracellular region, for example, via vesicles. Thus, it would be profoundly interesting to study the role of MLKL in circulatory system and immune regulation and its potential functions in immune-related diseases.

Some MLKL inhibitors have been discovered, however, these inhibitors still face many problems in terms of the druggability and clinical application. Future researches should focus on developing efficient, highly specific, bioavailable, and non-toxic MLKL inhibitors and exploring their applications in disease treatment.
